# Evolution and maintenance of mtDNA gene content across eukaryotes

**DOI:** 10.1042/BCJ20230415

**Published:** 2024-08-05

**Authors:** Shibani Veeraragavan, Maria Johansen, Iain G. Johnston

**Affiliations:** 1 Department of Mathematics, University of Bergen, Bergen, Norway; 2 Computational Biology Unit, University of Bergen, Bergen, Norway

**Keywords:** genome evolution, mitochondrial evolution, mtDNA

## Abstract

Across eukaryotes, most genes required for mitochondrial function have been transferred to, or otherwise acquired by, the nucleus. Encoding genes in the nucleus has many advantages. So why do mitochondria retain any genes at all? Why does the set of mtDNA genes vary so much across different species? And how do species maintain functionality in the mtDNA genes they do retain? In this review, we will discuss some possible answers to these questions, attempting a broad perspective across eukaryotes. We hope to cover some interesting features which may be less familiar from the perspective of particular species, including the ubiquity of recombination outside bilaterian animals, encrypted chainmail-like mtDNA, single genes split over multiple mtDNA chromosomes, triparental inheritance, gene transfer by grafting, gain of mtDNA recombination factors, social networks of mitochondria, and the role of mtDNA dysfunction in feeding the world. We will discuss a unifying picture where organismal ecology and gene-specific features together influence whether organism X retains mtDNA gene Y, and where ecology and development together determine which strategies, importantly including recombination, are used to maintain the mtDNA genes that are retained.

## Introduction

**Figure 1.**
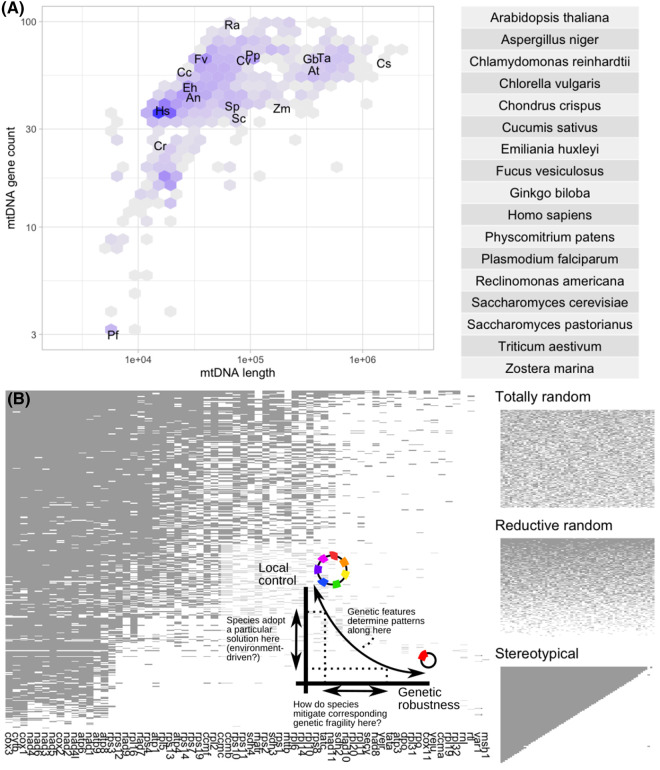
Genetic diversity in mtDNA. (**A**) Tiles show the number of samples in NCBI's Organelle Genome database with a given mtDNA length and gene count (darker colours denote more samples). Particular species of interest are labelled Xy, where X is the first letter of their genus and y the first letter of their species, with full names given in the box (for example, Hs is *Homo sapiens*). (**B**) Unique protein-coding mtDNA profiles, ordered by gene count, found in the NCBI Organelle Genome database. Each row is a unique profile (which may be observed in many individual species), each column is a gene, and dark pixels denote gene presence. Example profiles corresponding to completely random, random reductive, or completely stereotypical mtDNA evolution are shown on the right. The inset is a schematic of this article: retaining more or fewer genes may trade off local organelle control with genetic robustness, and species must maintain the genes they do retain against mutational hazard. Code to reproduce these figures is freely available at https://github.com/StochasticBiology/mt-gene-stats.

Mitochondria in most eukaryotes contain mitochondrial DNA (mtDNA). MtDNA encodes a subset of genes required for mitochondrial functionality. The particular set of encoded genes, the genetic organisation, and the physical structure of mtDNA vary dramatically across eukaryotes (Figure 1) [1,2]. MtDNA is inherited via diverse mechanisms across species, few of which resemble the inheritance of nuclear DNA [3–5]. Furthermore, the cellular ploidy and arrangement of mtDNA vary not just across species, but between cells and tissues and over development and time within individuals [6,7]. Table 1, in the spirit of the comprehensive graphical summary in [2], illustrates some of this diversity.

**Table 1.** Physical and structural diversity in mtDNA

Feature	Example values	Notes
Presence/absence	Simply absent from, for example, *Encephalitozoan cuniculi* and *Giardia*, *Entamoeba*, and *Trichomonas* (unicellular parasites)	
Structure	Linear, branched, circular, multichromosomal	
Copies per cell	Presumably >10^6^ in *Xenopus* oocytes, as 10^7^ mitochondria present Single nucleoid in many Apicomplexans (unicellular parasites)	[8]
Inheritance	Uniparental (maternal or paternal), biparental, doubly uniparental, uniparental with leakage, ‘triparental’ (from neither nuclear parent)	
Mutation rate	0.13 d_S_/mya *Pelargonium exstipulatum*; 2.53 × 10^−5^ d_S_/mya *Ceratozamia hildae* (flowering plants)	These values are only from plants, as cross-taxa comparisons can be complicated [9]
Protein-coding gene count	∼67 protein-coding genes (100 genes in total) *Andalucia godoyi* (jakobid protist) Two protein-coding genes *Chromella velia* (coral endosymbiont)	
Length	11.3 Mb *Silene conica* (flowering plant) 6 kb *Plasmodium falciparum* (unicellular parasite)	
Chromosome count	Single in many metazoans Hundreds in *Amoebidium parasiticum* (unicellular parasite)	
Different genetic codes	Vertebrate, yeast, protozoan, invertebrate, echinoderm, ascidian, alternative flatworm, chlorophycean, trematode, *Scenedesmus obliquus*, Thraustochytrium, Rhabdopleuridae	See (https://www.ncbi.nlm.nih.gov/Taxonomy/Utils/wprintgc.cgi)
Beyond above classification	*Trypanosoma brucei* mtDNA is partitioned into interlocking, chainmail-like ‘mini’ and ‘maxi’ circles; minicircles encode guide RNA to ‘decrypt’ the content of the maxicircles	

A summary of several aspects of mtDNA diversity from the references in this article, particularly inspired by Smith and Keeling [2] but with other data sources cited throughout this article.

MtDNA has downsides as a site for information storage. Replicating frequently, with a low effective population size, in an environment surrounded by potential mutagens, and with less packaging than nuclear DNA, the risk of mutational damage is high [10–14]. In some organisms (including most animals) mtDNA recombination is limited, raising the possibility of genome erosion via Muller's ratchet — the ongoing buildup of deleterious mutations until function is lost [15,16]. Maintaining high-ploidy mtDNA is likely costly [17] and raises possible conflicts between nuclear- and mtDNA-encoded genes [18].

Given these challenges, an obvious question is — why do organisms encode any genes at all in mtDNA? And the necessary corollary to any answer — how do organisms maintain the function of their encoded mtDNA genes? This review will attempt to describe some of the diversity of mtDNA behaviour through the lens of these questions (Figure 1B inset), attempting to provide a plausible and general set of principles that shape mtDNA evolution and maintenance across eukaryotes.

## Why do organisms encode any genes at all in mtDNA?

We must first consider the history of mitochondria. It is generally accepted that they were originally independent organisms — the closest known modern approximation to the ‘proto-mitochondrion’ is an α-proteobacterium [1,19–21]. Through an endosymbiotic event, the proto-mitochondrion was absorbed by a host — thought to be similar to an Asgard archaeon [1,22–24] — beginning the symbiosis that would give rise to modern eukaryotes [25–29]. An excellent overview of the subsequent changes in metabolic, regulatory, and import profiles is given in [1]; we will focus on the genome. Studies have attempted to reconstruct the properties of the proto-mitochondrion [30–33], with some work suggesting that it was originally an energy parasite [34]. The consistent picture is that it originally possessed the full complement of genes that a free-living organism would require.

Following endosymbiosis, redundancy with the host genome led to rapid loss of many of these genes [35,36]. Other genes were transferred to the host cell nucleus [19,37–39]. Several advantages have been proposed for nuclear encoding of mitochondrial machinery [40], with several focussing on the mutational hazard experienced by genes encoded in mtDNA [12,41] — which will be discussed in the contexts of different taxa throughout this article. These advantages include avoidance of Muller's ratchet (the inevitable buildup of deleterious mutations) [15,42,43], protection from damaging chemicals [10], enhanced capacity to fix beneficial mutations [40,42], and an energetic advantage over maintaining multiple mtDNA copies [17]. The physical transfer of mtDNA to the nucleus (giving rise to so-called nuclear mitochondrial sequences or NUMTs) is not a rare event [44,45], occurring over generational timescales in humans [46] and readily in plants [47]. However, the transfer of mtDNA is not the same as the transfer of functional gene content, as differences in genetic code (Table 1), regulation, and more must be addressed for functionalisation of transferred content. Several specific mechanisms for transfer have been discussed in detail [37,48,49], with increased recent focus on the properties of the intermediate state where a gene is contained in both nuclear and mtDNA [50,51].

These losses reduced the gene content of mtDNA dramatically, so that the most gene-rich mtDNAs discovered in modern eukaryotes have only dozens of genes, with the highest protein-coding gene counts so far found in jakobid protists *Andalucia godoyi* and *Reclinomonas americana* [52,53]. Overwhelmingly, the collection of genes found in modern eukaryotes are a subset of those in these gene-rich protists (Figure 1B) [38,54,55]. Reconstruction suggests that the last common ancestor of modern eukaryotes had a gene complement slightly larger than these jakobids [55]. Rare examples of mtDNA containing genes not found in these protists do exist. For example, octocoral mtDNA has acquired the *msh1* gene [56,57] — which we will meet again later — likely via virus-mediated horizontal gene transfer [58], and a restriction modification system has been acquired by the mitochondrion of a marine protist [59].

The physical structure of the mtDNA housing these genes is highly variable [2,60]. Many animal mtDNAs have a familiar circular structure, although mtDNA may form networks in human hearts [61], and mtDNA fragmentation is observed in lice [62] and cnidarians [63]. In contrast, plant and algal mitochondrial genomes are often split between many (often dozens of) different ‘subgenomic’ mtDNA molecules, each containing a subset of the full genome [64] and which may be linear or branched [65]. Linear mtDNA, including telomeres, is found across kingdoms [66,67]. Protist mtDNA structure exhibits substantial diversity [68], including branching and linear molecules, deviations from usual genetic codes [69], multiple chromosomes (sometimes with a single gene split across multiple mtDNA molecules and subsequently spliced together [70]), and the unusual ‘kinetoplast’ situation found in trypanosomes. Here, small ‘mini’ and large ‘maxi’ circles exist linked together in a ‘chainmail’ structure, with the minicircles encoding a guide RNA required to decode the mtDNA genome in the maxicircles [71].

Different eukaryotic kingdoms differ in both average number of mtDNA genes and the spread of gene count across different species (Figure 1B, Table 1, [38]). Focussing on the set of genes and not their ordering or arrangement (which does vary across species), animal mtDNA gene content is quite constant, with 13 protein-coding genes found across most animals. Exceptions to this complement include the aforementioned gain of *msh1* in corals [57] and some instances of loss in taxa including nematodes [72]. The gene content of many fungi often similar, and in many cases quite constant [50], although rearrangements and structural complexity can be dramatic (*cox1* in *Agaricus bisporus* contains 19 introns [73]). Plant mtDNA is generally more gene-rich and much more variable, with dozens of protein-coding genes and, often, substantial non-coding regions, which can range from 1% to >99% of the genome [74,75]. Across kingdoms, parasitism is often associated with reduced gene content [76]; in an extreme example, a cnidarian parasite retaining mitochondria but lacking mtDNA has been reported [77].

Among protists, gene profiles vary dramatically across different taxa [68]. Some unicellular parasites, with anaerobic lifestyles, have completely lost mtDNA [78–83]. Mitochondria that have undergone this — or even greater — reductive evolution are often referred to as mitochondrion-related organelles (MROs) including mitosomes and hydrogenosomes, depending on their particular metabolic properties. An anaerobic eukaryote without any organelle related to a mitochondrion has been reported [84]; reports of a dinoflagellate retaining aerobic mitochondria but lacking mtDNA [85] remain debated [86]. Other unicellular parasites, including many Apicomplexans, retain only three protein-coding genes *cox1*, *cox3*, *cob*; the related coral endosymbiont *Chromera* velia has additionally lost *cob* to retain only two protein-coding genes*.* On the other hand, the (also unicellular) jakobids above have the highest known mtDNA gene counts [52]. Different algae have markedly different profiles, with, for example, several dozen protein-coding genes retained by many red algae and some green algae retaining very few [87].

While not completely stereotypical, the genes retained across eukaryotic mtDNA are far from random [38,54] (Figure 1B). Several protein-coding genes, including *cox1*, *cox3*, *cob*, are retained in almost all species. Several specific *nad* and *atp* genes are also highly retained, while various *rps* and *rpl* genes are retained in a more limited and variable range of species. *sdh* genes, and a collection of others not encoding ETC subunits or ribosomal proteins, are retained by substantially fewer species [38,50,88]. Ribosomal RNA genes are consistently conserved (although often fragmented if ribosomal protein-coding genes are transferred from the organelle) [50]. Profiles of retained tRNA genes vary more substantially across taxa. A broad review is given in [89], which highlights some particular points of diversity. While many metazoans contain a complete, minimal set of tRNAs, other taxa vary substantially. Plant tRNA profiles are highly variable and rapidly evolving [90]; fungal profiles are also highly variable, with closely related species containing dramatically different sets. Trypanosome and alveolate mtDNA may completely lack tRNA genes, or contain a dramatically reduced set.

These observations turn our original question into two subquestions. First, what determines which *genes* are preferentially retained across species? And second, why does a particular *species* retain a given number of genes?

## Properties of a gene favouring retention in more species

The question of why a given gene is more or less likely to be retained in mtDNA has been discussed for decades. We focus here on mtDNA diversity across extant species; many endosymbiont genes were lost early post-endosymbiosis (presumably due in part to redundancy with the host) [1,35], and are not present in modern eukaryotes. One classic hypothesis for protein-coding genes relates to the hydrophobicity of a gene product [91,92]. It was first hypothesised that hydrophobic products, produced outside the mitochondrion, would be hard to import through the mitochondrial membrane to their required position. More recent research has suggested that hydrophobic products may be prone to mistargeting to the endoplasmic reticulum [91].

Another classic hypothesis is ‘colocation for redox regulation’ or CoRR [93,94]. Here, retaining genes local to the mitochondrion allows the individual organelle a tighter degree of local control over its redox function. This tighter control potentially allows faster, and more efficient, responses to new challenges — a change in bioenergetic demand or the degradation of key proteins, for example. Nuclear encoding makes it harder to fulfil the specific requirements of a given mitochondrion, out of the hundreds in the cell [94].

Other hypotheses have also been proposed. The economics — in the sense of the ATP budget for expression and maintenance — of organelle encoding has been argued to favour retention under some conditions [17]. It has been suggested that organelle genes can act as redox sensors, reporting the bioenergetic performance of a cell over time and facilitating control [95]. Issues with nuclear transfer and expression, including potential cytosolic toxicity of products [96] and differences in genetic code [40,97] have also been proposed to explain retention.

In an attempt to examine support for these hypotheses from an unbiased perspective, our group has used large-scale organelle genome data (thousands of eukaryotic mtDNA sequences and dozens of full nuclear genomes) with structural data and Bayesian model selection to identify likely features predicting the retention profile of a given gene [38,54]. We found that a combination of the hydrophobicity of a gene product and the GC content of the gene itself (independently of the general low GC bias in mtDNA [98,99]) robustly predicted (in unseen data) both whether a given gene would be retained in mtDNA or transferred to the nucleus, along with a signal associated with the pKa of the gene product.^1^
^1^The GC content of the mtDNA genome as a whole will certainly have changed over evolutionary time, and these studies accounted for the diversity of GC content baselines across different taxa. But it is the between-gene differences in GC content that was found — consistently across taxa — to predict between-gene differences in retention in this work. We also found that the ‘energetic centrality’ of a gene product — how physically central its position is in its containing complex — predicted mtDNA retention. Although correlations exist between these gene properties, their appearance together in the Bayesian model selection framework we used suggests that each provides independent power to predict retention. In contrast, features including molecular mass, energetic requirements for assembly, genetic code discrepancies and GC skew (G vs C usage) were not found to have any notable statistical support by this method. Although such an inference-based approach can only support and compare hypotheses statistically rather than directly test them experimentally (and can only consider the hypotheses with which it is presented), models based on these features predicted success of synthetic nuclear-mtDNA gene transfer experiments [88] (reviewed in [50]) and across other endosymbionts and organelles [100].

Why these features? The signal associated with hydrophobicity agrees with the hypothesis that difficulty in importing hydrophobic products — due to physical barriers and/or mistargeting — is a shaping factor. The energetic centrality of a product can intuitively — and explicitly [101,102] — be connected to its centrality in the assembly pathway of the complex. The control of complex assembly (in response to bioenergetic demand) in turn is a key determinant of redox regulation and therefore to CoRR [94].

GC content corresponds less readily to an established hypothesis. Following [103], we speculated that GC richness confers thermodynamic stability to a gene and therefore makes it more robust to the challenging environment of the mitochondrion. At a similarly speculative level, we proposed that ‘the synthesis of protein products enriched for higher-pKa amino acids may involve lower kinetic hurdles in the more alkaline pH of mitochondria…. favoring the retention of the corresponding genes’ [38]. Investigation of these hypotheses at a molecular level will be required to strengthen these arguments.

## Properties of a species favouring retention of more genes

Our dual question was why a given species is more or less likely to retain mtDNA genes. For example, parasitic species are expected to atrophy their mtDNA (and their mitochondria) both due to their reduced requirements for intrinsic energy transduction and due to their often low-oxygen environments [39,79,104–106]. Self-pollinating plants often transfer more genes to the nucleus than other plants; selfing has been shown theoretically to accelerate the transfer process when it confers an advantage [107,108]. More general theory across taxa has also been proposed. The ‘mutational hazard hypothesis’ proposes that mtDNA gene retention is safer in taxa with lower mtDNA mutation rates (for example, plants) [12,41]. A recent ‘burst-upon-drift’ model has been proposed to jointly explain variability in retention profiles and how nuclear transfer becomes fixed [50].

We recently hypothesised that the CoRR argument could connect species-specific demands on redox regulation to retention profiles more generally [109]. We considered a cellular model for the expression and degradation of organelle-targeted gene products, expressed either from oDNA (where high mutation rate poses a challenge) or the nucleus (where mutation is lower). We assessed the possible ‘supply’ of these products in the face of a ‘demand’ for organelle machinery imposed by the environment, which could be low and stable or high and highly varying. We found that in environments imposing a high and variable demand, the advantage of rapid supply from oDNA encoding outweighed the disadvantage of mutational hazard; the opposite was true in stable, facile environments. This theory predicts semi-quantitatively that more oDNA encoding is advantageous in organisms subject to strong, variable environmental demands, while nuclear transfer is advantageous in stable, less demanding environments.

This is supported by a cross-taxa phylogenetic comparative investigation of mtDNA gene count and ecology [76]. Here, attempting to account for the difficulty of comparisons across the broad, sparse, uncertain datasets available, we found fewer genes retained in organelles exposed to limited demands (endoparasites, and plastids without photosynthetic demands) and more genes in those exposed to more varying environments (in sessile organisms, deserts, and tropical oceans).

## Summary — why does organism x retain gene y?

It could never be claimed that these ideas give a complete answer to our first question. Indeed, it would be astonishing if a single, concise principle could explain all the diverse behaviour observed over billions of years of eukaryotic evolution. But the statistical treatments and connections to large-scale data above suggest that the proposed mechanisms do have some (not complete) explanatory power across a broad range of organisms. More genes are retained in mtDNA if species require tight local control of their redox machinery; properties of a gene including its product's hydrophobicity and centrality increase its propensity to be retained (Figure 1B inset). Overall, there would seem to be advantages to retaining genes in mtDNA in many cases. So…

## How do organisms maintain the function of the genes they retain in mtDNA?

### Mutational hazard

It is worth beginning by expanding on some issues associated with encoding information in mtDNA. MtDNA is less packaged and protected than nuclear DNA, frequently replicates, and its physical environment contains mutagens including the reactive oxygen species resulting from mitochondrial activity [10]. The contributions of these features to the accumulation of mtDNA damage is debated [110], with some evidence that oxidative damage may not be the dominant source of mutation [111]. Oxidative damage may be more like to induce strand breaks and abasic sites, and the specific behaviour of the polymerase gamma that replicates and proofreads mtDNA (including avoiding misreading damaged bases) also shapes mutational profiles [112–114]. Across these specific mechanisms, mutational hazard is clearly an issue [11–13], and can be directly demonstrated [115]. The limited number of genomes per cell limits the effective population size, potentially amplifying the effects of Muller's ratchet [14] and imposing a ‘drift barrier’ to the maintenance of efficient repair machinery [116]. [50] highlight that mutation rate does not provide a direct selective advantage for gene transfer at the level of the organism; however, it can readily be demonstrated that transfer is nonetheless evolutionarily favoured in populations (Supplementary Information).

Observed mtDNA mutation rates vary dramatically across taxa [9,12], between males and females [117,118], and between genes [119] — although such rates are a combination of a basal damage process and repair capacity, which also vary dramatically. In many animals, mtDNA mutation rates are well known to be higher than nuclear mutation rates. However, in plants [120], fungi [12], and indeed some animals (corals and sponges) [121,122], mtDNA mutation rates may in fact be lower than those in the nucleus. In plants [123], and more speculatively in these other taxa, mtDNA recombination-mediated repair will allow the correction of mutations [124–126], albeit at the cost of structural rearrangements of the genome [120,127] constituting an important mode of evolution [128].

The consequences of this mutational pressure on mtDNA are not homogeneous. Biochemical asymmetry (favouring hydrolytic deamination of cytosine) has the effect of favouring C → T conversion in mtDNA [98,99]. The GC content of mtDNA influences the free energy of the DNA duplex, suggested to influence mutational susceptibility of mtDNA [103].

**Figure 2.**
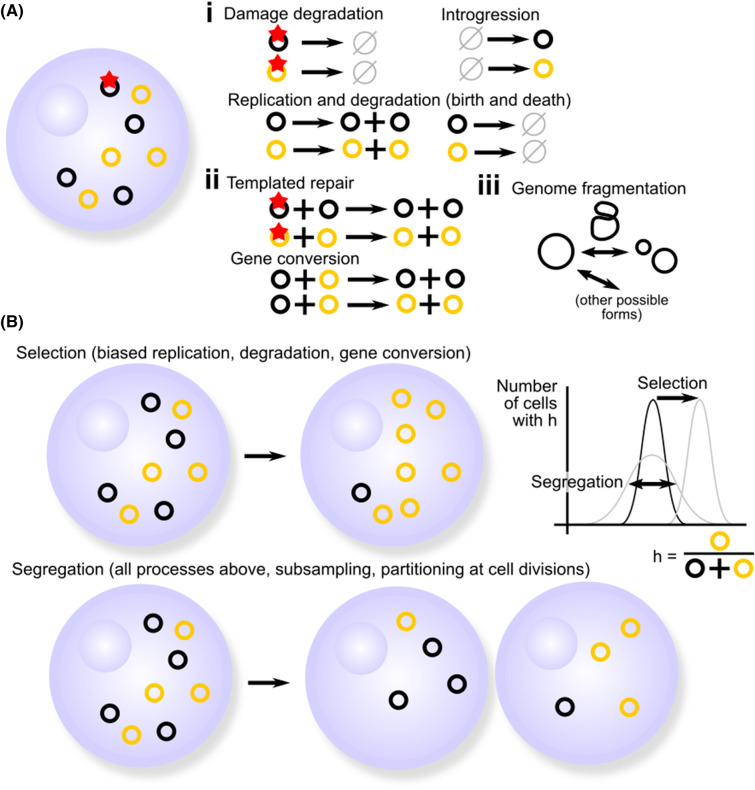
MtDNA-intrinsic processes shaping heteroplasmic mtDNA populations within cells. (**A**) Coarse-grained schematic of some processes that influence mtDNA populations, (i) independent of and (ii) dependent on recombination. Dark and light circles denote a general “heteroplasmic” picture of different mtDNA types; the star denotes molecular damage. (iii) Illustrates how recombination between regions of the same mtDNA molecule can lead to genome fragmentation and stoichiometric complexity. (**B**) Evolution of heteroplasmic populations viewed as selection and segregation processes. Selection shifts mean heteroplasmy, favouring one mtDNA type over another (due to type-specific differences between rates in (**A**)). Segregation increases (cell-to-cell) heteroplasmy variance without shifting the mean.

MtDNA mutations can be highly detrimental. Cells typically contain large (highly polyploid) populations of mtDNA molecules (Figure 2). The state where all these molecules have the same haplotype is termed ‘homoplasmic’; the converse, where at least two types exist, is ‘heteroplasmic’ [129–132]. Heteroplasmy, albeit on a small scale, is ubiquitous across many cell types and species [133–135]. In the case of two mtDNA types, the proportion of one (usually mutant) type is often referred to as the ‘heteroplasmy’ *h* of a sample, which could be a single cell, a tissue, or an organism^2^
^2^This terminology can be misleading, as if a mutant allele proportion exceeds 50% then heteroplasmy should arguably be redefined with respect to it as the major allele, but we will keep it for consistency with the literature. (Figure 2B). A nonlinear threshold effect is often observed, where a cell can support a heteroplasmic fraction of a dysfunctional mutant, but if this mutant frequency is too high then the cell experiences negative consequences [136]. This threshold allows mtDNA mutations to persist in populations, occasionally manifesting at high enough levels to cause disease [132].

As well as driving mitochondrial evolution across eukaryotes, mtDNA mutations have important translational consequences. Devastating human diseases arise when deleterious mtDNA mutations are inherited at high heteroplasmy [131,132] and understanding the organism-scale evolution of mtDNA is important in clinical approaches to address these diseases [137]. In plants, dysfunction due to mtDNA variants, while damaging for the organisms, can counterintuitively have very positive consequences for humans. ‘Cytoplasmic male sterility’ (CMS), arising from mtDNA or mitonuclear properties (see below), allows the easy production of hybrid crops, which often have substantially higher yields than inbred lines [138–140]. Although hard to precisely quantify, CMS is involved in a substantial proportion, or majority, of the global production of many tabletop crop species [140,141]. In this sense, ‘pathologies’ arising from plant mtDNA issues genuinely help feed the world.

### Intracellular competition and incompatibility between mtDNAs

An important parallel issue is the potential for competition between different mtDNA types within the same cell. There is some evidence that mtDNA heteroplasmy in and of itself is detrimental, even when no mtDNA types involved are deleterious [142–144].

Cell-to-cell distributions of heteroplasmy change over time in response to selection and segregation. Selection shifts the mean heteroplasmy over time; segregation increases the width of the cell-to-cell distribution (Figure 2B). Under various assumptions, the distribution of heteroplasmy has been shown [145] to correspond to population genetic solution in the absence [146] and presence [147] of selection. However, using this connection as suggested [145,148] to estimate selection and segregation rates from mtDNA measurements has several issues which recent statistical work has addressed [149]. Many other theoretical approaches have been used to explore the quantitative behaviour of heteroplasmy [150] including implementations of the Moran model [151] and Wright's models [152], classical models considering more specifics details of organelle genomes [153–156], and more detailed models including the roles of spatial structure and the microscopic processes involved [157–165].

Connected literature discusses selective differences between mtDNA types at this level as ‘segregation bias’ or ‘selfish proliferation’. Different mtDNA sequences may, for example, have different propensities for replication. A ‘replication–transcription switch’ has been proposed where favouring one process disfavours the other [166]. They may have different functional consequences for their host organelles and cells, so that selective pressures at those levels act to remove less functional types. A common picture is that an mtDNA type experiencing a replicative advantage is detrimental to cell, tissue, or organismal fitness. The different scales of selection in such cases can lead to proliferation (by replication) or removal (by removal of cells) of the selfish type [167–170]. Counterintuitively, physical properties of the system can lead to the proliferation of even deleterious mutations [159].

### Mitonuclear incompatibility

Another issue arising from the cellular context of mtDNA variation is mitonuclear incompatibility [18,171]. Because mitochondria require products encoded both by the nucleus and the mtDNA, it is possible for negative effects to arise from a combination of the nuclear and mtDNA alleles. A striking recent example is a lethal incompatibility affecting Complex I in naturally occurring hybrids [172]. Such interactions may drive speciation [173–175] and have been implicated in ageing [176], the evolution of sex [177,178], and shaping environment–gene and gene–gene interactions [179].

In cases where mtDNA is inherited maternally, the ‘mother's curse’ effect can lead to the accumulation of mutations which are damaging to males but are neutral or beneficial for females [180]. Presumably, if mtDNA is inherited strictly paternally, the comparable accumulation of mutations damaging to females but neutral or beneficial to males may occur — akin to the ‘father's curse’ picture [181]. Mitonuclear interactions are a mechanism by which these curses can be resolved [182]. As mitochondrial functionality relies on cooperation between nuclear- and mtDNA-encoded mitochondrial genes, the presence of a damaging mtDNA variant may induce strong selection for a nuclear allele that compensates this damaging effect [183]. Such ‘restorative’ nuclear variants are observed, for example, in CMS in plants (where male fertility, compromised by mtDNA variants, is restored by a nuclear factor) [184].

## General strategies for maintaining mtDNA function

Different cellular processes at the molecular, organelle, cellular, and organismal levels influence mtDNA evolution. Figure 2 gives a coarse-grained picture of some of the processes that shape cellular populations of mtDNA.

### Intracellular repair and removal

At the level of an individual mtDNA molecule, damage-repair mechanisms can be used to correct lesions, for example via fixing double-strand breaks or templating corrections by gene conversion [9,124,125,128,185]. At the level of organelles, if an mtDNA mutation corresponds to an organelle phenotype that can be individually sensed, cellular machinery can attempt to preferentially remove the mutant within that single cell via ‘mitophagy’ [186,187]. This within-cell process is part of mitochondrial ‘quality control’ [188–190].

### Intercellular removal

Between-cell selection can be used, removing whole cells if they contain an unacceptable proportion of the dysfunctional mutant. This scale of process is highly contingent on the broader context of a single cell. In a unicellular population, it simply corresponds to loss of less-fit individuals from the population. In a multicellular organism, it relies on the ability to remove cells, and is, therefore, more feasible in tissues with high rates of turnover than in quiescent tissues of static structure (for example, plant soma, animal brain, and muscle) [167,169].

**Figure 3.**
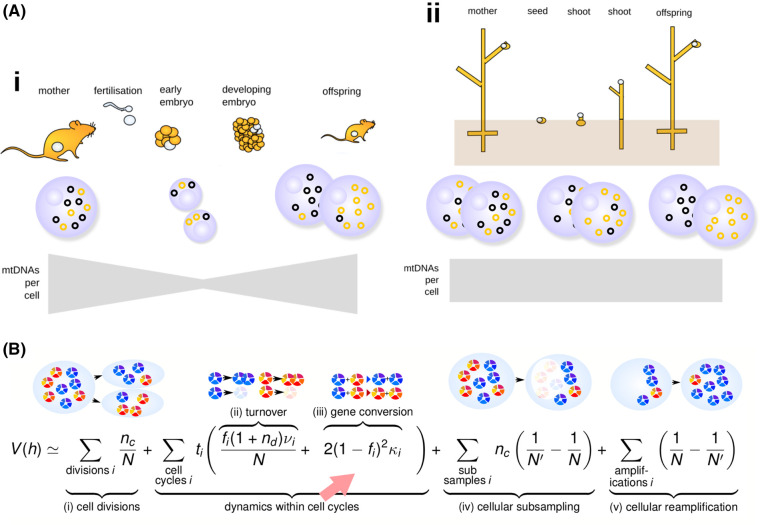
Segregation and developmental influences on mtDNA. (**A**) Illustration of mtDNA in the germline of (i) bilaterian animals (ii) plants. In (i), early developmental stages decrease mtDNA copy number per cell, subsampling the mtDNA population and imposing a physical ‘bottleneck’ that acts to accelerate drift due to other segregation processes. In (ii), a physical bottleneck is less pronounced or absent; segregation occurs due to other processes. (**B**) A mathematical model for segregation quantifies the heteroplasmy variance due to different processes [191]. All except gene conversion (arrowed) are amplified at low mtDNA copy number *N*; evidence suggests that animals employ turnover and partitioning (i, ii, iv–v) for segregation and plants make use of gene conversion (iii). Other pertinent parameters are f_i_ (fragmented mitochondrial proportion, linking physical and genetic behaviour) and ν_i_ (mitophagy rate); a full description can be found in the original paper.

In many organisms there is also a developmental axis to consider (Figure 3A). Depending on the germline structure of an organism, the timing and scale of selection can vary (for example, removing cells or embryos at different stages). For example, animal embryos containing (cells containing) a high mutant proportion may fail early developmental checkpoints and fail to develop further. The selection for mitochondrial quality, in the face of different mutational pressures, has been proposed to drive the evolution of a germline itself [192].

It is worth taking a second to disambiguate the various meanings that ‘selection’ can have in this context. Given the centrality of mtDNA to bioenergetics and eukaryotic life, it is almost self-evident that some mutations will be selected against (negative selection). Pathogenic human mtDNA mutations [132] and sterility-causing mutations in plants [193] are intuitive examples. However, it can be hard to identify at what level selection acts — intracellular, intercellular, and/or at the level of the organism itself [169,194,195]. In bulk samples, distinguishing intracellular and intercellular selection is challenging, as the same bulk dynamics would be observed for either level of selection. The dominant level may depend on circumstance: work in mice has suggested organelle-level selection [196], while recent single-cell work has found more support for intercellular selection in some circumstances [194]. Powerful theoretical work has demonstrated the capacity of selection at these different levels to maintain mtDNA through germline development [197]. Another subtle (and debated) question is the extent to which positive selection has shaped natural mtDNA populations. Can mtDNA diversity be explained by non-adaptive processes, including neutral ratchets [198], or must selection be invoked?

### Segregation

Any selection on or above the between-cell scale relies on there being diversity in heteroplasmy between cells. This ‘heteroplasmy variance’ (often written V(h)) is what intercellular or organismal selection can act upon to purify a population. The generation of V(h) is often referred to as ‘segregation’ or (particularly in the plant kingdom) ‘sorting out’. It can be achieved through various mechanisms (Figure 3) [191], and can occur in parallel with selection acting to change the heteroplasmy mean (Figure 2B) [160,197]. These include several process in Figure 2, including the random replication and degradation of mtDNA [157,161,199,200], the replication of a random subset of mtDNA molecules in a cell [201], random partitioning of mtDNA molecules at cell divisions [161,202–205], and gene conversion [191,206,207]. MtDNA sequence features partly determine segregation behaviour [208,209]. The physical distribution of mtDNA molecules in the mitochondrial population, which may be reticulated, fragmented, or a combination, shapes the segregation contribution of each of these processes [157,191,204,210] — the physical behaviour of mitochondria shapes the genetic segregation of mtDNA.

Segregation of deleterious mutations allows selection to remove entities (for example, individual cells, embryos, or organisms) in which a relatively high mutant load has been concentrated, leaving the remaining entities with lower mutant loads. This process can mitigate against Muller's ratchet because it allows descendant entities to inherit lower mutant loads than their ancestor. For example, average heteroplasmy amongst (surviving) offspring can be lower than in their mother — because high-heteroplasmy offspring did not survive. But segregation can also facilitate adaptation of beneficial mutations [211]. This is because fixing a new mtDNA type necessarily involves a heteroplasmic intermediate state (before all mitochondria in a cell harbour the new mitotype), and heteroplasmy can be detrimental even if neither mitotype is deleterious [142–144].

### Inheritance and exchange

The inheritance patterns of mtDNA in a given species contribute to its ability to maintain function and reduce genomic conflicts [5,181,212]. Strictly maternal inheritance avoids generating heteroplasmy by mixing parental mtDNA contributions, and hence limits the negative consequences of mixed mtDNA [142–144,213]. But in some circumstances an alternative may be desirable. If some paternal contribution is allowed, and recombination supported [4,5,214], heterozygosity can be maintained in a population and more rapid adaptation to changing environments may be supported [215]. Purely paternal inheritance, rarely observed (though more common in plastids [5]), has been suggested to support strong selection through a severe bottleneck [181,216].

Some species may support horizontal gene transfer of mtDNA on various scales, from the transfer of individual mitochondria (and hence mtDNA) between cells, to large-scale exchange of mtDNA content between individuals. Introgression — where mitochondrial content from another organism not involved in the nuclear reproductive process — has been naturally observed in algae [217], and is a key component of human therapies targeting the inheritance of mtDNA disease [137,218,219]. Grafting plants, an essential aspect of agriculture, can lead to introgression [47,220]. At the cellular level, transfer of mitochondria (and therefore mtDNA) between cells via tunnelling nanotubes has received substantial recent attention [221,222]. From a mathematical perspective, such cellular introgression can help stabilise evolving mtDNA populations [161,223] and has experimentally been found to rescue deleterious phenotypes [224,225].

Taken together, there are clearly a collection of different strategies that organisms can in principle employ to balance the priorities of maintaining existing mtDNA integrity and allowing adaptation to new conditions. We will now discuss how these possible strategies are employed by different eukaryotic species, and attempt to crystallise some principles underlying this diversity. Due to the vast amount of research on these topics, especially in vertebrates, we cannot hope to connect to every relevant study. Our goal is not (indeed, cannot be) to exhaustively survey all studied mtDNA behaviour, but rather to provide a combined general picture and specific examples of diversity across kingdoms. We hope to provide a summary picture and also (see Discussion) propose a mechanism whereby this summary can by expanded over time outside the confines of a single article.

## Specific strategies across eukaryotes

### Animals

MtDNA mutation rates vary across animals [226], with vertebrates often having mtDNA mutation rates 20× higher than nuclear rates, and other lineages (for example, corals) having very low rates [121]. Recombination in the mtDNA of many animals is usually thought to be limited, with evidence against rapid mtDNA recombination occurring in mice [227]. Evidence has been reported for recombination in mussels [228] and carp [229], and recent work in *Drosophila* has shown that recombination can repair double-strand breaks in mtDNA [230]. In human cell lines, mtDNA damage has been reported as being removed through degradation rather than repair mechanisms [231,232]. The existence of mitochondrial quality control through mitophagy in animals has been more established, and reviewed extensively (for example, [188,189]).

At the cellular level, animal mtDNA exhibits selection both in germline and somatic tissues. Favouring of one mtDNA type over another in somatic animal tissues over the lifespan of one organism has been observed over many model systems and many mtDNA pairings [169]. Mouse lines constructed to be heteroplasmic have been a common study model here [233], and all mouse tissue-specific patterns of selective advantage and disadvantage observed to date can be grouped on an overall ‘atlas’ of tissue profiles [169]. That work proposed an overarching explanation in terms of different degrees of ‘selfishness’ — different propensities to replicate rather than transcribe useful machinery — across different sequences. Selfish replication of mtDNA has also been the focus of study in other animal systems including nematodes [234] and flies [168] (as well as in other kingdoms, described in later sections). Different mtDNA haplotypes have been shown to have different respiratory behaviours in mice [235] and humans [236]. Nuclear factors shaping heteroplasmy in different mouse tissues have been reported [196,237,238] along with a role for mitochondrial fission–fusion balance [239]. Bodies of work have also explored the multi-level selection shaping mtDNA populations in, for example, nematodes [167,240]. In humans, tissue-specific selection is also observed [241], including for disease-causing variants [242]. Nuclear factors shaping such heteroplasmy evolution have been identified [243,244]. Many open questions remain, however, including the reasons why pathogenic variants experience clear negative selection in some tissues (for example, blood for the 3243 mutation in humans [242]) and not others, and the molecular mechanisms of mtDNA selection [245,246] remain incompletely understood.

Germline selection for mtDNA in animals has also been demonstrated, including in mice [247–250], flies [251,252], and humans [253]. Several mechanisms have been identified, involving nuclear factors [143] and mitophagy with mitochondrial fragmentation [251,252]. Selection through germline development, particularly at the intracellular level, can help purify mtDNA and avoid mutations proliferating during the establishment of the high-ploidy oocytes found in mammals [197]. Correspondingly, population-level evidence for mtDNA selection has been observed in humans [254,255]. Selective pressures acting at this broader scale have been proposed to involve gene expression profiles [256], transcriptional pressures shaping gene ordering [257] and environmental cues, for example, of temperature and altitude in humans [254,255,258], altitude in birds [259], and temperature and metabolism in fish [260,261].

Many animals exploit a developmental mechanism variously called the ‘germline bottleneck’ or ‘mitochondrial bottleneck’ to segregate mtDNA [130,262,263]. This mechanism typically couples a developmental reduction in mtDNA copy number per cell with random processes that segregate heteroplasmy between cells (Figure 3) [150,160]. In such animals, mtDNA copy number in oocytes is often high (for example, ∼2 × 10^5^ in mice [201,202,264]). During the first several cell divisions after fertilisation, this copy number per cell plummets to perhaps hundreds or thousands (the exact number is debated [202]) before being reamplified in the germ cells of the next generation. In parallel, random replication [200,201] and partitioning [202,203] generates cell-to-cell variability in heteroplasmy between developing germ cells, and hence between offspring [160,247]. This process, with different rates and numbers, occurs across bilaterians [150,265] including insects [266,267], humans [131,262,268], fish [269], and cattle, where it was originally observed [270,271]. Ongoing random replication of mtDNA continues this segregation throughout lifetimes [247,272]. Segregation also occurs in somatic tissue over time [240,269,273,274].

Several animals do not sequester a germline in the same way as vertebrates, including soft corals and sponges. Elegant theory work has connected this to the particular mutation pressures faced by these taxa (suggested to be relatively high background mutation rates and lower copying error rates), with the converse (low background mutation, high copying error) suggested to favour an early sequestered germline [192]. The absence of extreme mtDNA ploidy in these taxa and their modular growth plans have also been theoretically connected to their mtDNA maintenance [197]. Some members of these taxa, as mentioned above, have unusually acquired *msh1* in their mtDNA. Theory work has suggested that these two features may be connected, and that *msh1*-supported mtDNA recombination may assist segregation in the absence of a vertebrate-like germline bottleneck [191]. In some of these organisms, mitochondria are fragmented and highly motile, recalling structure and dynamics in plants (see next section) — for example, freshwater sponges [275].

MtDNA inheritance in animals is predominantly maternal. This is the case observed in humans; most claims against this rule [276] are controversial [277], and recent observations have indeed shown a lack of intact mtDNA in human sperm [278]. The extent of paternal leakage varies across animals; substantial leakage is observed, for example, in bees [279]. An exception to the maternal rule is the doubly uniparental inheritance observed in some bivalves [280–282]. The benefits and costs of the consequential paternal contribution to mtDNA in some individuals is the target of ongoing study [181,283].

### Plants

Mutation rates in plant mtDNA, while typically lower than nuclear mutation rates [12], vary dramatically across species [284] and are in part predicted by (somatic) genome copy number [9], in a relationship suggested to be linked to the availability of templates for repair. Plant mtDNA readily recombines [125,285–287]. This supports both homologous recombination-mediated damage-repair mechanisms [125,286,288–290] and gene conversion for templated repair [185] and segregation [207,291,292]. The relative plasticity of plant mtDNA has led to it being (rather unkindly) dubbed ‘the dumping ground’; a large amount of non-coding content, including material derived from the nucleus, plastid, and viral genomes is found in plant mtDNA [193,293,294]. The specific connection between recombination-driven mtDNA repair and genome evolution has been highlighted in [128,289,295].

As a consequence of this plasticity, the physical structure of plant mtDNA is both more complex and more variable than in animals [287,296,297]. The mtDNA genome is often spread over a collection of subgenomic mtDNA molecules [298, 299], and individual plant mitochondria typically contain less than a full genome [64]. Famous examples in the *Silene* genus involve the mtDNA genome partitioned into dozens of chromosomes, some of which contain no functional content [74,300]. These subgenomic molecules interact through recombination in a dynamic population [127,301,302], and individual mitochondria share mtDNA and its products through exchange on dynamic ‘social networks’ in the cell [141,298,299,303–305]. When *msh1*, responsible for organelle DNA maintenance, is perturbed, the dynamics of this social exchange are altered to support more mtDNA sharing [306]. Although less understood than in animals [307], quality control through mitophagy is established in plants [308–311] and likely serves to shape cellular mtDNA populations.

At the population level, the extent of selection on plant mtDNA has (like animals) been subject to debate [312]. MtDNA features clearly give rise to phenotypes that are detrimental to natural plants, including CMS. CMS involves the loss of male fertility which has been linked to mitonuclear interactions and both point mutations and structural rearrangements in mtDNA [139, 193, 313]. While detrimental to natural plants, CMS is of great use in agriculture, where sterile males support high-yielding hybrid production [138,140,141].

Non-chromosomal striping (NCS) is another example of selection linked to tissue-level differences in mitochondrial heteroplasmy. NCS is linked to deletions in mtDNA that impact the electron transport chain and has a more widespread impact on growth and development, including plant stature and yield in maize [314]. Tissue-level differences in heteroplasmy, possibly due to selective amplification of mtDNA fragments, have also been observed in tobacco [315] and rice [316]. Reduced nonsynonymous mutation in functional regions of genome has been reported in *Ginkgo* and rice [317] and even the selective neutrality of synonymous substitutions is debated, with some recent studies suggesting a role for selection [318].

Although known for over a century and foundational to organelle genetics [319], segregation in plants has classically been challenging to quantify, because the levels of heteroplasmy observed in naturally occurring plants was typically very low. Despite this, segregation has been reported in different taxa including carrot, olives, and *Silene* [320–322]. The existence and nature of a germline in plants is debated [323], and it does not seem to be the case that plants sequester an animal-like germline. Theory has explored the consequences of this for segregation mechanisms [191], finding that the increase in V(h) through gene conversion can proceed independently of cellular mtDNA copy number, and may, therefore, be a robust strategy in the absence of a physical mtDNA bottleneck.

To increase the quantitative understanding of plant segregation, recent work in Arabidopsis used an *msh1* mutant, in which *de novo* mtDNA (and cpDNA) mutations were readily generated [123]. Some heteroplasmic plants containing an admixture of these mutations and wildtype mtDNA were then back-crossed to the wildtype msh1, leading to plants with substantial heteroplasmy with either wildtype nuclear DNA or the *msh1* mutation. Heteroplasmy was tracked in these plants through development and between generations. Segregation was extremely rapid (an effective bottleneck size of ∼4) in the wildtype and seven times slower in the *msh1* mutant, pointing to a role for gene conversion in this rapid generation of V(h) [291,292]. Rapid segregation of plant mtDNA is likely to support ‘substoichiometric shifting’ (SSS), a process whereby an mtDNA type that is initially rare comes to dominate a sample [324–326].

Indirect evidence for the role of gene conversion in other plant species comes from a bioinformatic survey showing high expression of organelle recombination machinery in the shoot apical meristem (which will be responsible for producing sex cells) in barley, Medicago, rice, and potato [191]. In the shoot apical meristem (responsible for the aboveground germline), plant mitochondria physically meet in a network [327,328], which could support recombination more readily than the fragmented arrangement in other cell types [191]. In Zostera, powerful modelling work has combined individual and population-wide pictures to explore the roles of segregation and selection in shaping mtDNA [329,330].

Plants have long been observed to display a variety of mitochondrial inheritance strategies [331,332]. [5] provide an excellent review illustrating several of these, including maternal inheritance (common); maternal with paternal leakage (e.g. alfalfa [333]); paternal inheritance (e.g. cucumber [334]) and biparental inheritance (e.g. zonal geranium [335]).

### Fungi

Fungal mtDNA also has the capacity for recombination [191,336,337]. Evidence seems mixed on whether recombination occurs readily over organismal (as opposed to evolutionary) timescales, with some studies observing extensive recombination [338,339] and some with little observed [340]. Of course, the observation of recombination will depend on many features including species and the extent of heteroplasmy (as in plants, above).

In addition to random drift [341], various selective pressures have been shown to shape fungal mtDNA. A common example of ‘selfish’ mtDNA behaviour in yeast is the ‘petite’ mutant, harbouring a large-scale deletion that appears to confer a replicative advantage [342–344]. This mutant has been extensively studied, with over 100 nuclear factors shaping its evolutionary dynamics at the cellular level [345]. Recent single-molecule work has characterised the dynamics of generation and proliferation of this mutant, and its link to recombination hotspots in the mtDNA genome [346].

The proliferation of different mtDNA types in fungi in response to different environmental pressures has been observed across species, including for fungicide treatments [347,348], salinity [349], and host species [350] and mtDNA type has been shown to confer temperature tolerance [351]. The action of multi-level selection, within- and between cells, has been characterised in budding yeast [352], with roles for mitochondrial fission and mitophagy identified in shaping heteroplasmic populations [353].

In unicellular organisms, the behaviour of mtDNA at cell divisions determines (largely) mtDNA segregation and (completely) the inheritance of mtDNA [354–356]. The physical process of mtDNA segregation at cell divisions in unicellular fungi has been studied in depth [204], with evidence that yeast controls the partitioning of mtDNA at divisions more tightly than binomial partitioning. Yeast mtDNA inheritance is biparental [3], but selective inheritance of particular mtDNA types has long been observed [344]. In hybrid situations a colony can come to favour one paternal type through preferential (and environmentally determined) retention [357]. Other fungi, including the multicellular *Neurospora crassa*, exhibit uniparental inheritance and segregation of artificial heteroplasmy over time [358]. Across the kingdom, a range of inheritance and segregation behaviours are observed [336,337]

### Protists

Presence of recombination machinery varies across protists [191], but many species have highly fragmented mtDNA genomes that might suggest recombination-mediated coupled [2,68]. Minicircles, almost corresponding to individual mtDNA genes, have been recently reported in red algae [359]. The euglenozoan Diplonema papillatum has multiple small mtDNA fragments smaller than the size of individual genes, which must be spliced together from these fragments [70]. Recent work dramatically increasing the sampling of protist mtDNA has revealed genome plasticity reminiscent of the plant kingdom in stramenopiles [68].

In several protists, a single mitochondrion with a single mtDNA nucleoid exists per cell [360]. The physical segregation machinery has been characterised in the unusual case of trypanosomes [361]. In multicellular protist species, segregation is not to our knowledge well explored. Multicellular algae can have relatively complex developmental plans, somewhat reminiscent of plants, that could conceivably harbour comparable segregation processes [362]. In an interesting parallel to the case of green plants above, ultrastructural analysis has found mitochondria in a brown alga to be generally fragmented except in female gametophytes (perhaps analogous to the reticulated mitochondria in the plant shoot apical meristem) [363].

Instances of external pressures shaping protist mtDNA are as diverse as the species in this section. Heteroplasmy profiles in *Fucus* have been observed to depend on geography [364]. Selective pressures acting on trypanosome mtDNA have been suggested to include intrinsic factors like translational efficiency and transcript cost [365], and it has been found that mtDNA is essential for the parasite's transmission stage [366]. An interesting branch of research has drawn parallels between mitochondrial disease in Dictyostelium and other taxa, finding that heteroplasmic mtDNA gene disruption has systemic effects on organism physiology [367,368].

Inheritance patterns in protists are as diverse as the species involved. In some slime moulds, mtDNA inheritance has been reported as uniparental [369]. In various marine algae, maternal, paternal, and heteroplasmic mtDNA inheritance has been observed (reviewed in [370]) — including maternal, paternal, and biparental modes within one *Porphyra* (Rhodophyta) species [371]. An unusual mechanism of triparental inheritance — where mtDNA is inherited from a cell that is neither of the (biparental) nuclear parents — has been observed in Dictyostelium [372] (recalling the artificial introduction of mtDNA from a third-party donor in mitochondrial replacement therapies [137,218,219]).

## Discussion

### A synthesis of observations and theories

**Figure 4.**
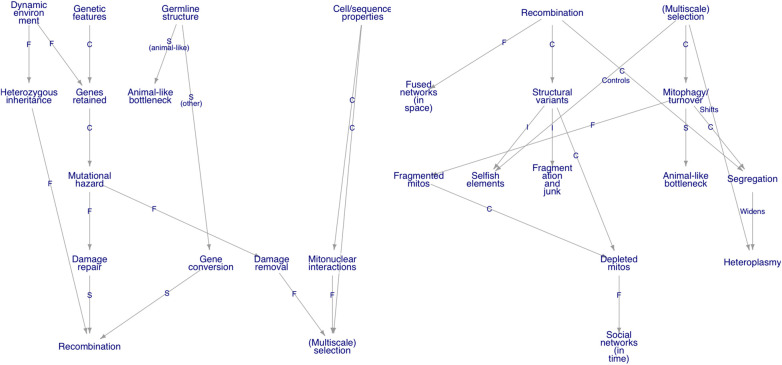
Knowledge graph-style synthesis of mtDNA influences. An outline of the (non-exhaustive) set of influences on coarse-grained mtDNA structure that we have discussed. Nodes are concepts; edges denote links between concepts, labelled including with C, causes; F, favours; S, supports; I, includes. (Left) external factors affecting the poise of recombination and multiscale selection processes acting on mtDNA. (Right) the consequences of these processes for mtDNA behaviour. Code to reproduce this figure is freely available at https://github.com/StochasticBiology/mt-gene-stats.

Having surveyed at least some of the diversity of mtDNA content and behaviour across eukaryotes, are we better placed to answer our original questions? We can at least attempt to synthesise some of the observations we have noted (Figure 4).

The first clear observation is that the textbook picture of an isolated mammalian mitochondrion with a non-recombining, 16 kb circular mtDNA encoding 13 proteins is unrepresentative of eukaryotes. Gene retention, physical structure, inheritance, and mutational hazard varies hugely across species. Given the similarities in process and machinery to bacterial recombination, mtDNA recombination is likely ancestral (discussed, for example, in [9]) and plays varied roles across kingdoms in repair and segregation of damage. Structural, genetic, and stoichiometric complexity result.

A path through the knowledge graph in Figure 4 can be used to summarise some of the principles in this article. A combination of the physical features of individual genes [38,88] and the challenges faced by mitochondria in an individual species together (and non-exclusively) influence mtDNA gene retention profiles (Figure 1B inset). Strong, dynamic environmental changes favour gene retention for CoRR [76,93,109]. Maintaining mtDNA heterozygosity to adapt to changing environments may also influence which inheritance patterns are favoured [211,215], and the necessity of dealing with differing mutational pressures and maintaining mtDNA may influence germline timing and properties [192,197].

The requirements for repairing consequent mtDNA damage then influence to what extent to mtDNA recombination may be usefully employed by a species. An organism's developmental germline profile also seems to affect whether recombination is used to segregate damage [191] or an animal-like bottleneck strategy of high ploidy is used [192,197]. As mtDNA molecules must physically meet to recombine, the physical dynamics of mitochondria also shape the genetic activity of recombination [191,304,306]. Multiscale mtDNA removal, at the organelle, cellular, or organismal levels, also contributes to damage control and function maintenance. The recombination benefits of templated repair and segregation via gene conversion are balanced by the structural variance induced by recombination, which can lead to genome fragmentation, junk inclusion, and the appearance of selfish elements [2,287].

### Across eukaryotes — across organelles?

Many of the arguments outlined above do not particularly require the organelle of interest to be a mitochondrion. We found that the same features of hydrophobicity, GC content, and energetic centrality predict cpDNA gene retention as well as mtDNA retention — and, strikingly, this prediction is quantitative in the sense that a model trained on mtDNA retention profiles predicts cpDNA retention profiles [38]. The theory developed suggesting that strong and dynamic environmental demands favour organelle gene retention also applies to cpDNA [109], and we observed consistencies among environmental features statistically linked with gene retention profiles in both organelles [76]. Indeed, a weak but robust correlation between mtDNA and cpDNA gene counts is detectable in the subset of species for which records are available for both [373]. Symmetry particularly in sets of genes encoding ribosomal proteins in mtDNA and cpDNA has been observed [102]. CpDNA heteroplasmy appears to sorted rapidly and with similar drivers to mtDNA in plants [291,374]. However, the link is perhaps better founded on the left hand side of Figure 4 than the right hand side. The physical embedding of mtDNA and cpDNA can be very different. In plants, mitochondria contain less than a full genome copy [64] and continually meet to exchange contents. Chloroplasts contain many genome copies and are not known to exchange cpDNA [127], so the physical and ‘social’ dynamics described above are likely not comparable.

Beyond chloroplasts, hydrophobicity is also linked to the gene profiles of other endosymbionts [14], including the photosynthetic endosymbiont acquired more recently in *Paulinella* algae [375,376], the nitroplast [100], numerous endosymbiotic bacteria in insects [14], and other symbiotic bacteria [38]. It is tempting to speculate — though not without caution [2] — that these principles may constitute universal modulators of endosymbiont-organelle genome evolution.

### An ongoing synthesis?

Any attempt to describe phenomena across all eukaryotes will necessarily be incomplete. We would like to do two things that are perhaps somewhat unusual. First, we offer our sincere apologies to the authors of studies which are aligned with the topic of this review which we have missed a connection with. In no cases was this deliberate and the corresponding author would (always!) appreciate suggestions of aligned literature. Second, we propose a public document where comments on the manuscript, suggestions of related content, and other aligned messages can be posted. This document can be found here https://tinyurl.com/mtdna-review, and readers should be able to post comments freely and anonymously. We will synthesise content and comments on the Github repository associated with this paper https://github.com/StochasticBiology/mt-gene-stats.

## Abbreviations

CMS: cytoplasmic male sterility
MROs: mitochondrion-related organelles
mtDNA: mitochondrial DNA
NCS: non-chromosomal striping
SSS: substoichiometric shifting
