# Supplementary material for: Evolution and maintenance of mtDNA gene content across eukaryotes
Source: Biochem J. 2024 Aug 5;481(15):1015–42. doi: 10.1042/BCJ20230415 (PMC11346449; doi:10.1042/BCJ20230415)
Supplement: Supplementary Material [file BCJ-481-1015-s1.pdf]

Supplementary Information

To demonstrate how mutational hazard can stablilise transfer of genes to the nucleus, we consider a simple toy model. We simulate a population of *N* organisms evolving through non-overlapping, asexual generations. A single gene determines fitness. It can be encoded in the mitochondrion or in the nucleus. If in the mitochondrion, it experiences a loss of function mutation with probability μ per genome per generation, which leads to a reduction in fitness. If in the nucleus, it never mutates. The simulation begins with a single individual with nuclear encoding and *N-1* with organelle encoding. Roulette wheel selection is used to construct a new generation given the fitnesses of the previous generation, and the proportion of individuals with the gene encoded in the nucleus is reported after t = 100 generations. Supp. Fig. 1 shows the results for *N*=100 with different fitness effects of the mutated gene, and 10^4 instances of each parameterisation. As μ increases, the proportion of nuclear-encoding individuals increases above the neutral case of 1/*N* towards unity. There is no contribution of mutation rate to the fitness function: it suffices that a lineage prone to mutation is more likely to die out. Code to reproduce this analysis is freely available at https://github.com/StochasticBiology/mt-gene-stats.

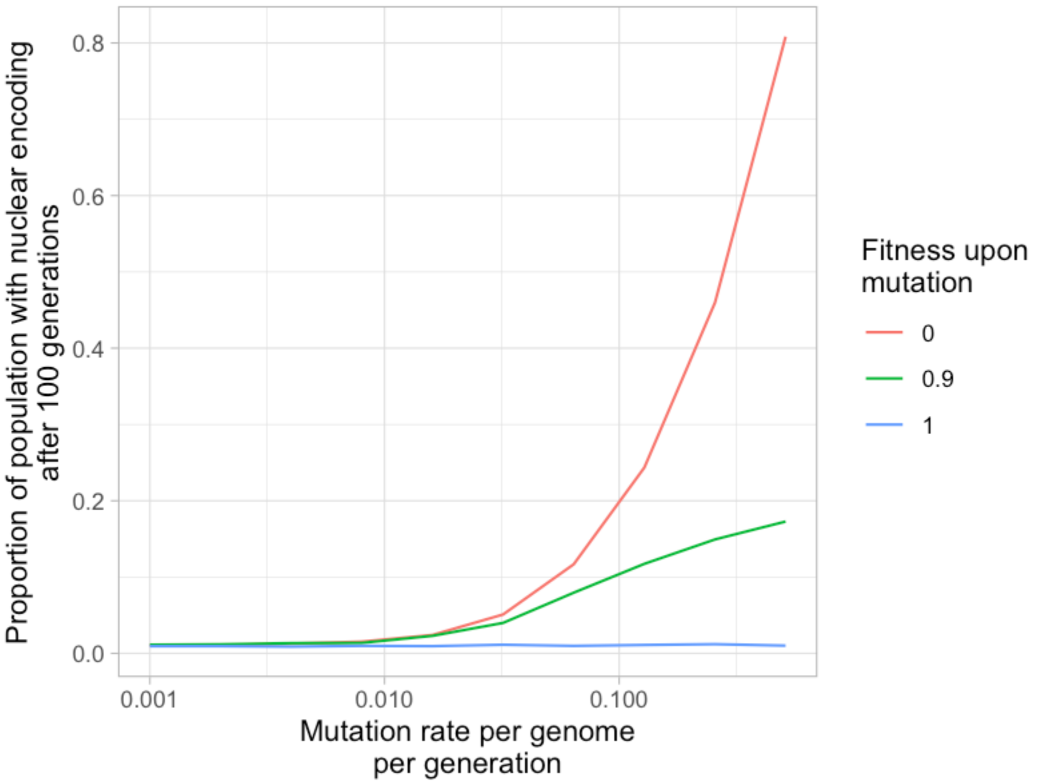

Supplementary Figure 1. Nuclear encoding of a gene is preferred under higher organelle mutation rates as individuals harbouring deleterious mutations are removed from the population.
